# A distance-based kernel for classification via Support Vector Machines

**DOI:** 10.3389/frai.2024.1287875

**Published:** 2024-02-26

**Authors:** Nazhir Amaya-Tejera, Margarita Gamarra, Jorge I. Vélez, Eduardo Zurek

**Affiliations:** ^1^Department of Computer Science, Universidad del Norte, Barranquilla, Colombia; ^2^Department of Industrial Engineering, Universidad del Norte, Barranquilla, Colombia

**Keywords:** support vector machines (SVMs), classification, distance-based kernel, kernel method, machine learning, supervised learning

## Abstract

Support Vector Machines (SVMs) are a type of supervised machine learning algorithm widely used for classification tasks. In contrast to traditional methods that split the data into separate training and testing sets, here we propose an innovative approach where subsets of the original data are randomly selected to train the model multiple times. This iterative training process aims to identify a representative data subset, leading to improved inferences about the population. Additionally, we introduce a novel distance-based kernel specifically designed for binary-type features based on a similarity matrix that efficiently handles both binary and multi-class classification problems. Computational experiments on publicly available datasets of varying sizes demonstrate that our proposed method significantly outperforms existing approaches in terms of classification accuracy. Furthermore, the distance-based kernel achieves superior performance compared to other well-known kernels from the literature and those used in previous studies on the same datasets. These findings validate the effectiveness of our proposed classification method and distance-based kernel for SVMs. By leveraging random subset selection and a unique kernel design, we achieve notable improvements in classification accuracy. These results have significant implications for diverse classification problems in Machine Learning and data analysis.

## 1 Introduction

Support vector machines (SVMs), proposed to solve binary classification problems and further extended for regression (Cortes and Vapnik, 1995; Wang et al., 2021), grouping, and multiclass classification problems (Vapnik, 1995, 1998), are a supervised machine learning (ML) technique used in the field of artificial intelligence and data mining to solve classification and regression problems (Boser et al., 1992; Cortes and Vapnik, 1995).

SVMs offer greater flexibility and better performance in high-dimensionality problems, and their performance in classification problems is comparable to other ML algorithms (Roy et al., 2015). The classical SVMs are based on finding a hyperplane in a higher dimensional space that can optimally separate the different classes of data (Vapnik, 1995, 1998). To deal with high dimensionality, SVMs base their operation on the use of a kernel function. A kernel is a mathematical function that transforms high-dimensional input data into a higher-dimensional space in which an optimal hyperplane effectively separates the data. The optimal hyperplane is a geometric surface, which can be a boundary line or a plane, and depends on the type of kernel and the number of variables (i.e., features). Once this hyperplane has been found, it can be used to predict the class of new data.

Different research has been conducted to improve and obtain better classification results, including either different variations of SVMs or proposing a new kernels. Several types kernels are available in the literature and can be used in SVMs, each with different properties and applications. Some common examples of kernels include the linear kernel, the polynomial kernel, and the radial basis kernel. Choosing the right kernel depends on the problem and requires a certain amount of experimentation and testing to find the one that best suits the needs. In addition to choosing an appropriate kernel for the data, the computational complexity in SVMs is *O*(*n*^3^) (Cervantes et al., 2008). This constitutes one of the main drawbacks when developing and validating predictive models based on this ML algorithm, and limits their application to big data sets.

In this article, we propose (1) a new distance-based kernel for a SVM classifier and (2) an innovative method to obtain the best subset of the original data that achieves the maximum accuracy in the testing data. In contrast to classical ML, we randomly selecting subsets of the training data that have great potential for correctly classifying instances over the test data, while making sure that the size of the training data set is smaller than the size of the test data. Furthermore, instead of applying the kernel on the full training data set, usually 70 or 80% of the sample size, we compute a distance matrix from the small training subset and use it as the kernel in SVM. This approach offers great computational advantages both in the training stage and in the final implementation. The paper is organized as follows. Section 2 presents the theoretical background, Section 3 the related work, Section 4 describes our proposal, and in Section 5 we present our results. Finally, in Section 6 we present our conclusions and discussion.

## 2 Background

### 2.1 SVMs

In SVM, a distinction is made between linearly separable and non-linearly separable cases. Given a set of elements represented by the tuple (*x*_*i*_, *y*_*i*_) where *x*_*i*_ are the vectors containing the features and *y*_*i*_ the classes for *x*_*i*_. In the case of having two classes and if all the elements can be correctly separated, then the dataset is said to be separable. Given a separable set: *X* = {*x*_*i*_, *y*_*i*_} where *i* = 1, ⋯ , *n*, *x*_*i*_∈ℝ and *y*_*i*_∈{−1, 1}. A separation hyperplane is a linear function capable of separating said function without error given by *H*(*x*_*i*_) = (*w*_1_*x*_1_+⋯+*w*_*n*_*x*_*n*_)+*b*(*w, x*_*i*_). However, it is not always possible to design a linear function that allows to separate all the cases correctly. Hence, the solution is to create a representation or mapping of the dataset to a space with a higher number of dimensions, known as feature space. In this space, a linear function can be designed and used for classification and that can be expressed by ϕ = *X*→*R*^*n*^. The element ϕ can be a very complex function, but it is not necessary to know or calculate the function since, a kernel can express it.

In the feature space defined by ϕ, $f\left(x\right)=w^{T}\phi \left(x_{i}\right)$ is obtained. The Lagrangian dual function of *f*(*x*) can expressed as $L_{d}=\sum a_{i}-\;\frac{1}{2}\sum a_{i}a_{j}y_{i}y_{j}\left[\phi \left(x_{i}\right)\cdot \phi \left(x_{j}\right)\right]$, where *a*_*i*_ and *a*_*j*_ denotes the Lagrange multipliers associated with the support vectors, and *y*_*i*_ and *y*_*j*_ are the class labels. The kernel trick is embedded in the expression [ϕ(*x*_*i*_)·ϕ(*x*_*j*_)]. The dot product of the feature vectors in the transformed space is expressed implicitly through the kernel function *K*(*x*_*i*_, *x*_*j*_) avoiding the need to explicitly compute the transformation ϕ. In other words, *K*(*x*_*i*_, *x*_*j*_) is a kernel function that calculates the dot product of the transformed feature vectors without explicitly calculating the transformed vectors. Therefore, *L*_*d*_ incorporates the kernel trick to efficiently handle high-dimensional feature spaces, making SVMs computationally more tractable.

The kernel function is essential in SVMs to perform the ML process, being the linear and polynomial kernels the most common. The linear kernel is effective when the data are linearly separable (Cortes and Vapnik, 1995), and is represented by $K\left(x_{i},x_{j}\right)=x_{i}^{T}x_{j}$. The polynomial kernel, on the other hand, allows introducing non-linearity in the feature space and is given by $K\left(x_{i},x_{j}\right)={\left(\gamma x_{i}^{T}x_{j}+r\right)}^{d}$, where *x*_*i*_ and *x*_*j*_ are the feature vectors, γ is a parameter that controls the influence of the linear part of the kernel, *r* is the intercept term, and *d* is the degree of the polynomial. Thus, data that are not linearly separable can be separable in the original space can be separated using this kernel. If *d* = 1, the polynomial kernel resembles a linear kernel. However, as *d* increases, the ability to model non-linear relationships in the input data also increases (Cortes and Vapnik, 1995).

A characteristic of the SVM algorithm is the use a regularization parameter known as *C*, which influences the penalty that is applied to the error when training (James et al., 2013). If the *C* parameter is too low, it can result in underfitting; if *C* is low, the algorithm chooses a hyperplane with a higher training error but good generalization, which this is known as a smooth hyperplane. On the other hand, when *C* is high, the algorithm selects a hyperplane with a lower training error, making it a better fit for the training data, and when *C* is set too high, it can lead to overfitting.

Let **x** = (*x*_1_, ⋯ , *x*_*n*_) and **y** = (*y*_1_, ⋯ , *y*_*n*_) be binary vectors representing the predicted and data classes of *n* individuals or instances, respectively. By comparing *x* and *y*, a confusion matrix can be computed as in Table 1A. Each instance can be classified into one of the following categories: (*a*) True Positive or TP, when it is correctly classified as “Yes”; (*b*) False Positive or FP, when it is incorrectly classified as “Yes”; (*c*) False Negative or FN, when it is incorrectly classified as “No”; and (*d*) True Negative or TN, when it is correctly classified as “No”. Note that, in an ideal scenario, FP and FN would be zero.

**Table 1:** Confusion matrices for **(A)** binary and **(B)** multiclass classification problems.

**(A) Confusion matrix for binary classification**					
**Predicted class**	**Data class**				
	**Yes**		**No**		
Yes	*a*, TP		*b*, FP		
No	*c*, FN		*d*, TN		
**(B) Confusion matrix for multiclass classification**					
**Predicted class**	**Data class**				
	**1**	**2**	**3**	**4**	**5**
1	*n*_11_	*n*_12_	*n*_13_	*n*_14_	*n*_15_
2	*n*_21_	*n*_22_	*n*_23_	*n*_24_	*n*_25_
3	*n*_31_	*n*_32_	*n*_33_	*n*_34_	*n*_35_
4	*n*_41_	*n*_32_	*n*_43_	*n*_44_	*n*_45_
5	*n*_51_	*n*_32_	*n*_53_	*n*_44_	*n*_55_

In (B), five classes are present in the data.

For more than two classes, that is, for a multiclass classification problem (Hossinm and Sulaiman, 2015), the confusion matrix is shown in Table 1B. Let *n*_*ii*_ and *n*_*ij*_ be the number of instances correctly classified in class *i*, and the number of instances in class *j* classified in class *i*, respectively. Under this notation, the total number of correctly classified instances is $\overset{k}{\underset{i=1}{\sum }}n_{ii}$, the FN for class *i* is $\overset{k}{\underset{i=1}{\sum }}n_{ij}$, *i* > *j*, and the FP for class *i* is $\overset{k}{\underset{i=1}{\sum }}n_{ij}$, *i*<*j*. Of course, the total number of instances is $T=\overset{k}{\underset{i=1}{\sum }}\overset{k}{\underset{j=\;1}{\sum }}n_{ij}.$

### 2.2 Performance metrics

Based on the values of TP, TN, FN, and FP, it is possible to assess the performance of a binary classifier using different metrics. Table 2 shows the most commonly used performance metrics in binary classification problems, their formulas and description.

**Table 2:** Performance metrics commonly used in binary classification.

**Metric**	**Formula**	**Description**
Accuracy (ACC)	$\frac{TP+TN}{T}$	Describes the ratio of correct predictions to the total number of predictions made. Higher values are better.
Sensitivity (SEN), recall or true positive rate (TPR)	$\frac{TP}{TP+FN}$	It represents the ability of a model to correctly identify positive instances. Higher values are better.
Specificity (SPE) or true negative rate (TNR)	$\frac{TN}{TN+FP}$	It represents the ability to correctly identify negative cases among all the actual negative instances. Higher values are better.
Precision or positive predictive value (PPV)	$\frac{TP}{TP+FP}$	It indicates the proportion of true positives regarding all the elements identified as positive. Higher values are better.
Negative predictive value (NPV)	$\frac{TN}{TN+FN}$	Proportion of true negatives in relation to all predictions that were evaluated as negative. Lower values are better.
False discovery rate (FDR)	$\frac{FP}{FP+TP}$	Proportion of incorrect positive results among all predicted positive results. Lower values are better.
False negative rate (FNR)	$\frac{FN}{TP+FN}$	Proportion of positive cases that were incorrectly labeled as negative in relation to the total number of positive cases. Lower values are better.
False positive rate (FPR)	$\frac{FP}{FP+TN}$	Proportion of negative instances that are incorrectly classified as positive. Lower values are better.
Lift	$\frac{TP/\left(TP+FP\right)}{\left(TP+FN\right)/N}$	Ratio between the response rate obtained by the model and the average response rate in the general population. It represents the harmonic average of Precision and Sensitivity metrics. Indicates whether the model's accuracy is compromised in datasets with uneven distribution. Higher values are better.
*F*1-score	$\frac{2\cdot TP}{2\cdot TP+FP+FN}$	Combines the precision and recall measures into a single value. Higher values are better.

*T*, total number of instances.

If the number of classes is *k* > 2, the confusion matrix is of dimensions *k*× *k* as in Table 1B. For class *i*, the elements on the main diagonal are the correctly classified elements, and the elements outside the main diagonal are the incorrectly classified elements. The values of *TP*_*i*_, *FP*_*i*_, *FN*_*i*_ and *TN*_*i*_ can be determined as follows: *TP*_*i*_ are the elements of class *i* that is part of the main diagonal; *FP*_*i*_ is the sum of column *i*, excluding the *TP*_*i*_ value; *FN*_*i*_ is the sum of the values in row *i*, excluding the *TP*_*i*_ value; *TN*_*i*_ is the sum of the values of all rows and columns, excluding row *i* and column *i*. In multiclass classification, we calculate the ACC as the ratio between correctly classified elements and all classifications. It is also possible to compute other metrics using macro-averaging, where a measure is the average of the same measure for all classes (Sokolova and Lapalme, 2009).

### 2.3 Distance and similarity metrics

A distance is a mathematical function *d*(*x, y*) where all *x, y* ∈ *X* and fulfills the following properties (Deza and Deza, 2013):


$$\begin{array}{l} \left(\text{i}\right)\;d\left(x,y\right)\geq \;0;\\ \left(\text{ii}\right)\;d\left(x,y\right)=\;d\left(y,x\right);\\ \left(\text{iii}\right)\;d\left(x,x\right)=0;\;and\\ \left(\text{iv}\right)\;d\left(x,z\right)\leq d\left(x,y\right)+d\left(y,z\;\right). \end{array}$$

To assess the similarity between data sets of binary nature, the Jaccard index, the Jaccard distance, and the Rogers and Tanimoto (1960), and Sokal and Michener (1958) similarity coefficients are commonly used (Table 3). The Jaccard index compares two data sets and determines how many members are common in the sets and which are dissimilar; the Jaccard distance is based on the Jaccard index and represents a measure of dissimilarity. On the other hand, Rogers-Tanimoto coefficient, is useful to penalize false positives on binary sets. Finally, the Sokal-Michener coefficient is calculated by dividing the intersection of the sets by the sum of their sizes plus their intersection (Table 3). To obtain a measure of dissimilarity between two instances **x** and **y** with *n* binary attributes, we subtract the value of the similarity coefficient from 1. Thus, values closer to 0 indicate greater similarity, values closer to 1 indicate greater dissimilarity, and a value of one indicates that the two data sets have no elements in common.

**Table 3:** Similarity measures.

**Name**	**Formula^a^**
Jaccard similarity index (Jaccard, 1901)	$I_{j}\left(x,y\right)=\frac{p}{p+q+r}$
Jaccard distance	*D*_*j*_(*x, y*) = 1−*I*_*j*_(*x, y*)
Rogers-Tanimoto coefficient (Rogers and Tanimoto, 1960)	$D_{RT}\left(x,y\right)=\frac{p+s}{p+s+2\left(q+r\right)}$
Sokal-Michener coefficient (Sokal and Michener, 1958)	$D_{SM}\left(x,y\right)=\frac{p+s}{p+q+r+s}$

^a^Here, *p* is the number of attributes where *x*_*k*_ = *y*_*k*_ = 1, *q* is the number of instances where *x*_*k*_ = 1 and *y*_*k*_ = 0, *r* is the number of attributes where *x*_*k*_ = 0 and *y*_*k*_ = 1, and *s* is the number of attributes where *x*_*k*_ = *y*_*k*_ = 0.

## 3 Related work

Considerable work has been conducted for binary classification and kernels using SVMs. For instance, Zhang et al. (2022) use a SVM model called DB-SVM to predict N6-methyladenine DNA modification. DB-SVM takes a sequence alignment and generates a distance matrix, which is used as the kernel. The authors reached >92% a level of accuracy in two independent data sets of different nature. Despite the satisfactory results, the evaluation of the DB-SVM model is limited to only two data sets.

Based on the Kernel Ridge Regression (KRR), Hazarika et al. (2021), proposed the Kernel Ridge Regression Based on Intuitionistic Fuzzy Membership (IFKRR) for binary classification. In this method, each training sample is assigned an intuitionistic fuzzy number to determine its membership. The degree of membership is given by the distance to the center of the corresponding class, while the degree of non-membership is given by the ratio between the number of heterogeneous points and the total number of points in their neighborhood. These authors also proposed the Affinity-based Fuzzy KRR (AFKRR) (Hazarika and Gupta, 2023) model for binary classification with unbalanced data. However, neither of these proposals do not consider multiclass classification.

One significant drawback of SVMs is the latency in their response (Zhou, 2021). An attempt to enhance response speed involves creating subsets of the original data through clustering techniques before applying SVMs (Gao et al., 2019). However, this strategy may suffer from poor performance, as the generated groups might overfit due to class imbalance. To tackle this issue, Fayed and Atiya (2021) propose a supervised clustering technique that aims to achieve almost balanced groups. The main concept involves dividing the densest class into *k* clusters, identifying those closest to the decision boundary, and expanding them to incorporate the closest patterns from all classes. This ensures an equal representation of both classes. Although this approach has shown satisfactory results compared to SVM, Random Forest, and Adaboost (Fayed and Atiya, 2021), it currently cannot handle multiclass classification.

On the other hand, it has also been suggested to combine SVMs with other techniques to tackle highly complex tasks. For example, Zhang et al. (2019) propose an approach to identify weed species in crop fields using machine vision and SVMs. First, the Grabcut algorithm and *K*-means clustering are used to remove the background and segment the weed images. Further, Local Weighted Maximum Margin Discriminant Analysis (LWMDP) is utilized to extract highly discriminative features from the images, taking advantage of both local and class information from the data. Finally, a SVM is used to identify week species, achieving remarkable accuracy levels.

Other studies in the literature use publicly available data sets to perform classification based on several features (i.e., variables). These data sets include (*i*) the Cleveland Heart Disease (Cleveland HD) (Janosi et al., 1989), which are 13 features and 303 instances; (*ii*) the divorce data set (Mustafa Yntem, 2019) includes 170 instances (0: non-divorce, *n* = 86; 1: divorce, *n* = 84) representing responses to a total of 54 questions/features valued on a scale ranging from 0 to 4; (*iii*) the Spambase data set (Mark Hopkins, 1999) is a collection of 4,601 instances (1,813 spam and 2,788 non-spam instances) and 57 attributes; (*iv*) the Rice dataset (Koklu and Cinar, 2019b) has 3,810 instances classifying two species of rice grains (*n* = 2,180, Osmancik species; *n* = 1,630, Cammeo species) and seven attributes representing different morphological characteristics of the rice grains extracted from images obtained through image processing (Koklu and Cinar, 2019a); and (*v*) the Banknote dataset (Lohweg, 2012), which was derived from 1,372 images of both genuine (55%) and counterfeit (45%) banknotes and measured four distinct attributes. These data sets are publicly accessible from the UCI machine learning repository (Markelle et al., n.d.) and will further be used to evaluate our method and compare our results with those previously reported.

Latha and Jeeva (2019) used the Cleveland HD data set and fitted an ensemble of multiple classifiers to increase accuracy. In this ensemble, the authors included Boosting, Bagging, Stacking and Majority vote, while using Bayes Network, Naive Bayes, Random Forest, C4.5, Multilayer Perceptron and Part as classification techniques (Latha and Jeeva, 2019). By utilizing this ensemble, the total accuracy improved between 5.94 and 7.26%, with the Majority vote achieving the best accuracy. Feature selection techniques have also been applied to improve accuracy for this data set. Verma et al. (2016) proposed a hybrid data mining model. Using clinical data, a selection of features is conducted using Correlation-based Feature Selection (CFS) and Particle Swam Optimization (PSO). Then, supervised learning algorithms such as multi-layer perceptron (MLP), multinomial logistic regression (MLR), fuzzy unordered rule induction algorithm (FURIA) and C4.5 were integrated into the hybrid model. This hybrid model achieved a 90.28% accuracy when applied to the Cleveland HD data set.

Similarly, Alotaibi (2019) used RapidMiner (https://rapidminer.com/) as well as five different machine ML (i.e., Naive Bayes, Decision Tree, Random Forest, Logistic Regression and SVM). These models are cross validated with 10 iterations. The results reveal that the Decision Tree model reaches an accuracy of 93.19%, followed by SVM with 92.30%. The Random Forest model obtains an accuracy of 89.17%, while Logistic Regression and Naive Bayes obtain 87.36 and 87.27% accuracy, respectively. Khan et al. (2017) used the Weka (https://www.cs.waikato.ac.nz/ml/weka/) to study several classification techniques using this data set. The authors applied a specialized algorithm for feature selection, while the instances with missing data were eliminated to ensure the integrity of the analysis (Khan et al., 2017). Techniques such as RIPPER, Decision Tree, Artificial Neural Networks (ANNs) and SVMs were used. The results show that SVM reaches 84.12% accuracy, followed by RIPPER with 81.08%, ANNs with 80.96%, and Decision Tree with 79.05%.

Using the divorce data set (Mustafa Yntem, 2019), Sharma et al. (2021) implemented several classifiers (i.e., Perceptron, Decision Tree, Random Forest, Naive Bayes, K-NN and SVMs) to predict divorce cases. SVM was applied with a tolerance value of 0.001, and a polynomial kernel with 3 degrees of freedom. The results revealed that the Perceptron classifier achieved the highest accuracy with a 98.53% using a 60/40 proportion for the training and testing data. Interestingly, with these same proportions, accuracy values between 95.59 and 98.53% were achieved for the remaining classifiers. Simanjuntak et al. (2020) used the Reverse Propagation Neural Network (BPNN) algorithm. For a deeper evaluation, they contrasted the results by implementing various feature selection techniques (i.e., Information Gain, Gain Ratio, Relief-F and Correlation). Without feature selection, the authors reached an accuracy of 98.24%; the accuracy ranged between 98.82 and 99.41% when feature selection was applied. Juarez-Lopez et al. (2021) present a comparative analysis of C4.5, JRip, *K*-NN and SVM with a linear kernel, while using the full data set and CFS. The authors used 2/3 of the full data set for training and 1/3 for testing (Juarez-Lopez et al., 2021).

Ghosh and Senthilrajan (2023) used the Spambase data set (Mark Hopkins, 1999) to develop a framework for email evaluation. The research focuses on the comparison of the performance of 13 different classifiers, including SVMs, considering a set of only eight attributes. According to their results, Random Forest achieves the highest accuracy with 99.93%, while Naïve Bayes achieves the lowest, with an accuracy of 79.53%.

On the other hand, Ilhan et al. (2021) used Deep Neural Networks (DNNs) to classify the rice varieties present in the Rice data set. The data were normalized to improve the performance of multilayer neural networks; the authors used 10-fold cross-validation and achieved an average accuracy of 93.04%. Similarly, Koklu and Cinar (2019a) conducted a study to evaluate the performance of various classification techniques, including Logistic Regression, MLP, SVM, Decision Trees, Random Forests, Naïve Bayes and *K*-NN. All available features were used for evaluation and 10-fold cross-validation approach was used for all models. The best performance was obtained with Logistic Regression (93.02% accuracy), while the lowest performance was achieved with *K*-NN (88.58% accuracy).

Finally, Yadav et al. (2021) used six classification techniques (i.e., SVM, Random Forest, Logistic Regression, Naïve Bayes, Decision Tree, and *K*-NN) to discriminate banknotes using the Banknote dataset (Lohweg, 2012). The authors considered three combinations of training/testing proportions (i.e., 80/20, 70/30, and 60/40). Through a feature selection process, all features were found to be important. For the 80/20 percentage, *K*-NN achieved the highest accuracy at 100%, while Naïve Bayes had the lowest accuracy at 84%.

### 3.1 Proposed method

SVM training can be slow due to factors such as large data size. To address this challenge, we proposed, instead of using the complete dataset in the training process as other research similar proposal using SVM suggest (Li et al., 2014, 2023; Dudzik et al., 2022), to randomly select smaller data subsets through multiple iterations. This iterative approach seeks to identify representative data subsets that capture the essence of the total set, which, in turn, seeks to facilitate the classification process. This proposal has no limitations on the number of features or instances in the dataset. As a result of this method, the training is performed with smaller subsets of data but seeks to obtain high classification performance.

**Figure 1:**
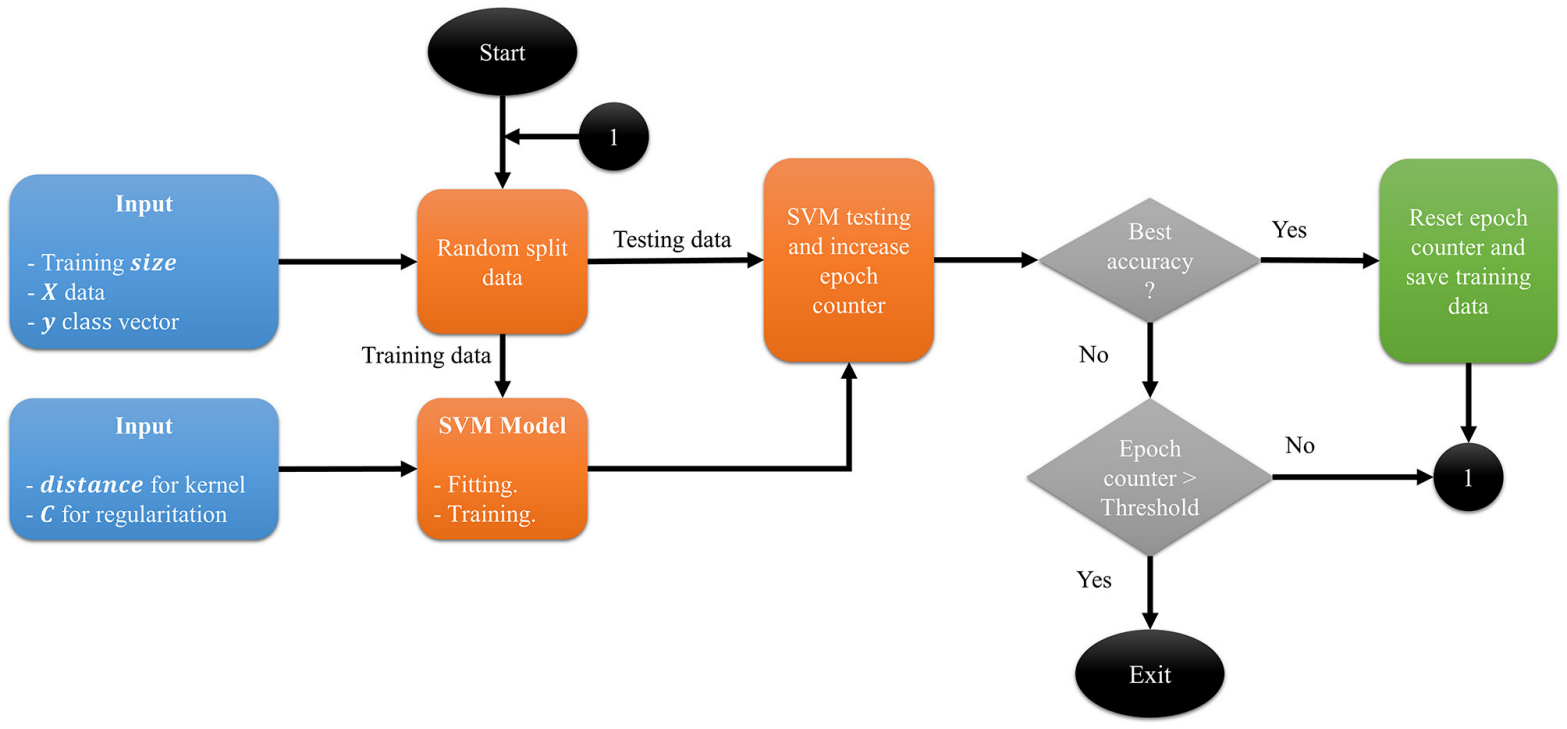
Diagram process. The inputs are the data *X*, the classification vector *y*, the size of training set, the value of the *C* parameter and the name of the distance for the kernel.

The proposed method, presented in Algorithm 1, is an SVM with a kernel based on dissimilarity measures and is given by *K*(*x, y*) = 1−*dissimilarity*_*matrix*(*x*_*i*_, *x*_*j*_, *distance*). Initially, the data must be preprocessed and converted to binary data, and the response vector *Y* must only have two classes. Next, the five arguments for the proposed method are defined: (1) *X*, a binary data set of dimensions *n*×*m*; (2) a classification vector *Y* of size *n*×1; (3) *N*, the value of the testing size; (4) the value of the regularization parameter *C*, which influences the penalty applied to the error during the training process; and (5) a distance or dissimilarity metric used by the proposed kernel. These five arguments correspond to the inputs to the process (Figure 1), and we save *X*_*train*_ if a better accuracy is obtained or until the process finishes. Of note, the distance or dissimilarity metric must be selected according to the nature of the binary data (for some options, see Table 3).

**Algorithm 1:**
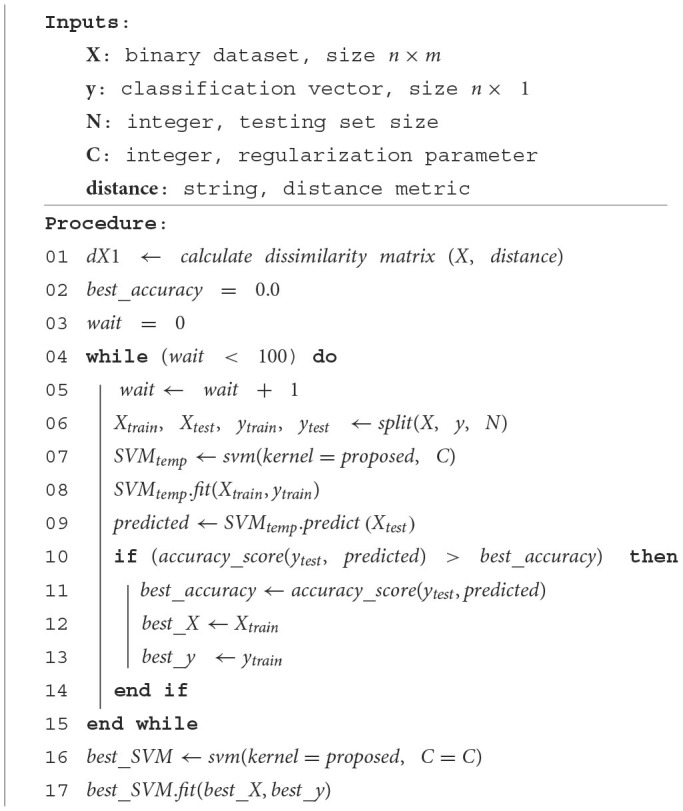
Main procedure.

Figure 1 shows the process diagram. From the initial data set *X*, the dissimilarity matrix is calculated. The result is a square matrix of size *n*×*n*. To improve the visual representation of the information in this square matrix, we use multidimensional scaling (MDS), a statistical technique used for dimensionality reduction and exploratory data analysis using the dissimilarity between instances (Borg et al., 2013). When MDS is applied with two components, a matrix of dimension *n*×2 is obtained, which can be used for further analysis, including visualization.

First, the proposed method starts by creating two variables called *best*_*accuracy* = 0 and *wait* = 0. In a repetitive cycle that will be executed up to 100 times, our goal is to find the best set of the training data that generates the *best*_*accuracy*, while respecting the percentage split of training and testing entered by the user. Interestingly, an heuristic analysis where tests were conducted with different numbers of runs showed that, for a greater number of iterations in the repetitive cycle, the execution time increased but no improvement in accuracy was observed.

The variable *wait* is increased by one unit and the following procedure is performed. Starting from *X* and *Y* the training and testing subsets are randomly generated, that is, *X*_*train*_, *Y*_*train*_, *X*_*test*_ and *Y*_*test*_ are created. With these subsets, an instance SVM is created using the proposed kernel and the parameter *C*. Next, the model is fitted using *X*_*train*_ and *Y*_*train*_, and predictions are made with *X*_*test*_. At this stage, we check the prediction accuracy; if it improves, we assign the variable *wait* a value of zero, update the value of the variable *best*_*accuracy*, and retain the values of the parameters *C*, *X*_*train*_, and *Y*_*train*_. If the *wait* counter is below the defined threshold, *X*_*train*_, *Y*_*train*_, *X*_*test*_ and *Y*_*test*_ are randomly generated once again. If the *best*_*accuracy* is maintained for the defined threshold, the iterations are terminated.

At the end of the iterations, the algorithm saves the values of *C*, *X*_*train* and *Y*_*train* exhibiting the best accuracy when using *X*_*test* and *Y*_*test*. Based on the value of *C* and the proposed kernel, a SVM classifier is generated, which will be called *best*_*SVM* and will be adjusted with *X*_*train* and *Y*_*train*. *best*_*SVM* is the classifier with better accuracy in training and will be used to make predictions. From each of these predictions the corresponding metrics are calculated to evaluate their performance.

## 4 Numerical experiments

### 4.1 Datasets

Table 4 lists the datasets used for the validation of the proposed method. All datasets are publicly accessible in the UCI machine learning repository (Markelle et al., n.d.). Additional information about these data sets is presented in Section 3.

**Table 4:** Datasets used and the number of attributes before and after preprocessing.

**Dataset**	**Attributes**	**Instances**	**Attribute type**	**Attributes after binarization**
Cleveland heart disease	13	303	Categorical, integer, real	37
Divorce	54	170	Integer	216
Spambase	57	4,601	Integer, real	169
Rice	8	3,810	Real	21
Banknote	5	1,372	Real	12

Of note, to protect privacy, the attributes in the Spambase dataset (Mark Hopkins, 1999) do not contain actual message information but instead indicate the frequencies of certain words and characters, the number of uppercase letters in the message, among other characteristics. On the other hand, attributes include in the Rice dataset (Koklu and Cinar, 2019b) include information about area, perimeter, major axis length, minor axis length, eccentricity, convex area, and extent of the grains. It is worth noting that these attributes were extracted from images obtained through image processing (Koklu and Cinar, 2019a). Table 5 presents the relevant characteristics of the Cleveland HD dataset. The classification column vector contains values from 0 to 4, values from 1 to 4 are different cardiovascular disease (CVD) risks, and value 0 is no CVD risk.

**Table 5:** Description of features in the Cleveland HD dataset.

**Feature**	**Description**	**Range of values**
age	Age in years	29–77
sex	Sex (1 male, 0 female)	0–1
cp	Chest pain type (1 = typical angina, 2 = atypical angina, 3 = non-anginal pain, 4 = asymptomatic)	1–4
trestbps	Resting blood pressure (in mm Hg on admission to the hospital)	94–200
chol	Serum cholesterol in mg/dl	126–564
fbs	Fasting blood sugar > 120 mg/dl (0 = false, 1 = true)	0–1
restecg	Resting electrocardiography results (0 = normal, 1= ST-T wave abnormality, 2 = showing probable or definite left ventricular hypertrophy by Estes' criteria)	0–2
thalach	Maximum heart rate achieved	71–202
exang	Exercise induced angina (0 = no, 1 = yes)	0–1
oldpeak	ST depression induced by exercise relative to rest	0–6.2
slope	The slope of the peak exercise ST segment (1 =upsloping, 2 = flat, 3 = downsloping)	1–3
ca	Number of major vessels (0–3) colored by fluoroscopy	0–3
thal	Thallium scan (3 = normal, 6 = fixed defect, 7 = reversable defect)	3–7
num	Class attribute	0–1

### 4.2 Data preprocessing

This section describes the procedure applied to each dataset with the aim of transforming its attributes into a binary representation, which will allow its use in accordance with the technique proposed in this article. The dimensions of each data set after binarization are specified in Table 4.

The Cleveland HD dataset contains 303 instances, siz contain missing values from the time of testing. We removed the instances with missing values, leaving a total of 297 instances. Categorical features were converted into “dummy variables”. Thus, each numerical feature was converted into categorical variables by generating three ranges from the original feature, hence splitting the values into three parts, to avoid creating numerous additional values for each feature. The resulting dataset is further used to test binary classification and multiclass classification. When performing binary classification, the values in the class vector, which range from 0 to 4 are grouped into two categories: the original values of 0 remain unchanged, while the original values of 1–4 are grouped into category 1. For multiclass classification, the original class is used. The features after preprocessing are summarized in Table 6.

**Table 6:** Description of features in the Cleveland HD dataset after preprocessing.

**Feature**	**Dummy variables generated**	**Description**
age	3	age < 45, 45 < age < 66, age >66
sex	2	0 = female, 1 = male
cp	4	1 = typical angina, 2 = atypical angina, 3 = non-anginal pain, 4 = asymptomatic
trestbps	3	trestbps < 129, 129 < trestbps < 164, trestbps > 164
chol	3	chol < 272, 272 < chol < 418, chol > 418
fbs	2	0 = false, 1 = true
restecg	3	0 = normal, 1= ST-T wave abnormality, 2 = showing probable or definite left ventricular hypertrophy by Estes' criteria
thalach	3	thalach < 114, 114 < thalach < 157, thalach > 157
exang	2	0 = no, 1 = yes
oldpeak	3	oldpeak < 2, 2 < oldpeak < 4, oldpeak > 4
slope	3	1 = upsloping, 2 = flat, 3 = downsloping
ca	3	ca < 1, 1 < ca < 2, ca > 2
thal	3	3 = normal, 6 = fixed defect, 7 = reversable defect

The Spambase, Rice, and Banknote datasets are distinguished by their continuous numerical attributes. To use the proposed method in this article, it is necessary to appropriately binarize these attributes. The binarization process was conducted as follows: a parameter called “bins” is set, determining the number of ranges into which each column of the dataset will be divided. Using the minimum and maximum values of each column, the range limits are defined, ensuring that the total range is evenly divided into the specified number of bins. Subsequently, binarization is applied to each column of the dataset using the previously calculated ranges. As a result of this process, a new dataset is generated, consisting of binary columns corresponding to each bin. This procedure enables the transformation of continuous numerical attributes into binary columns, thereby facilitating their subsequent analysis and the application of specific techniques within the context of this study.

Finally, the Divorce dataset is characterized by features on a Likert scale, ranging from 0 to 4. To align these attributes with the proposed method, a binarization process is performed. In this case, it is unnecessary to establish range limits, as the possible values for each attribute are predetermined and discrete. Consequently, each attribute is transformed into a series of binary columns, with each column representing one of the potential attribute values. Thus, binarization is accomplished by assigning a value of 1 to the column corresponding to the specific attribute value, while the columns corresponding to other potential values are assigned a value of 0.

### 4.3 Computational experiments

The process described in Algorithm 1 is ran iteratively by combining the different parameters. All experiments were performed in Python version 3.9.2 using NumPy and sklearn libraries. Because of the nature of our data (i.e., binary data), we used the implementations of the Jaccard, Rogers-Tanimoto and Sokal-Michener distances (Table 3) in the sklearn library. In our experiments, the size of the test data was *N* = {60%, 70%, 80%, 90%} of the original data, and the values of the regularization parameter for the SVM were *C* = {10, 20, 30, 40, 50, 60, 70, 80, 90, 100}. As a result, for each dataset, a total of 120 parameter combinations (*N*, *C*, and kernel configuration) for were considered.

## 5 Results

This section presents the results obtained by implementing the proposed method and varying its parameters. The classification capability of the proposed approach was evaluated on the training and test data set and calculating several metrics. Tables 7, 8 present the different combinations of *N* and *C* that allow us to obtain the best performance in the training and testing processes, respectively. For example, value 40/20 in the third row and third column of Table 7 represents the size testing data set as a percentage of the full data set (*N* = 40%) and the parameter *C* (*C* = 20). The next row corresponds to the achieved metric (*ACC* = 1.000) according to the selected distance/dissimilarity. Similarly, Table 8 presents the different combinations of *N* values and *C* that allow us to obtain a greater predictive capacity in the testing process.

**Table 7:** Combinations *N*/*C* (*N* is the test size in percentage, and *C* is the parameter that controls the penalty for classification errors) resulting in the best possible performance for our proposal on the subset of training data.

**Dataset**	**Combination**	**Performance measure**							
	**Distance**	**ACC**	**SEN**	**SPE**	**PPV**	**NPV**	**FDR**	**FNR**	**Lift**
Cleveland HD	*N*/*C*	40/20	40/20	40/20	40/20	40/20	40/20	40/20	10/20
	Jaccard	1.000	1.000	1.000	1.000	1.000	0.000	0.000	2.636
	*N*/*C*	20/60	20/60	20/60	20/60	20/60	20/60	20/60	20/60
	Roger-Tanimoto	1.000	1.000	1.000	1.000	1.000	0.000	0.000	2.458
	*N*/*C*	40/50	40/50	40/50	40/50	40/50	40/50	40/50	40/50
	Sokal-Michener	1.000	1.000	1.000	1.000	1.000	0.000	0.000	2.145
Divorce	*N*/*C*	40/10	40/10	40/10	40/10	40/10	40/10	40/10	10/70
	Jaccard	1.000	1.000	1.000	1.000	1.000	0.000	0.000	3.400
	*N*/*C*	10/60	10/60	10/60	10/60	10/60	10/60	10/60	10/60
	Roger-Tanimoto	1.000	1.000	1.000	1.000	1.000	0.000	0.000	3.400
	*N*/*C*	20/90	20/90	20/90	20/90	20/90	20/90	20/90	20/90
	Sokal-Michener	1.000	1.000	1.000	1.000	1.000	0.000	0.000	2.615
Spambase	*N*/*C*	10/80	10/80	10/80	10/80	10/80	10/80	10/80	10/100
	Jaccard	1.000	1.000	1.000	1.000	1.000	0.000	0.000	1.749
	*N*/*C*	10/90	10/90	10/90	10/90	10/90	10/90	10/90	10/100
	Roger-Tanimoto	0.984	0.972	0.992	0.988	0.982	0.011	0.027	1.707
	*N*/*C*	10/100	10/100	10/90	10/90	10/100	10/90	10/100	10/100
	Sokal-Michener	0.989	0.984	0.996	0.993	0.988	0.006	0.015	1.679
Rice	*N*/*C*	30/60	20/80	30/60	30/60	10/90	30/60	20/80	10/20
	Jaccard	0.872	0.833	0.909	0.923	0.855	0.076	0.116	2.214
	*N*/*C*	40/100	40/50	10/100	10/100	40/50	10/100	40/50	20/40
	Roger-Tanimoto	0.872	0.881	0.914	0.943	0.844	0.056	0.118	2.199
	*N*/*C*	40/30	20/100	10/60	10/60	20/20	10/60	20/100	10/100
	Sokal-Michener	0.870	0.870	0.929	0.934	0.847	0.065	0.129	2.172
Banknote	*N*/*C*	40/60	10/40	40/80	40/100	10/20	40/100	10/40	40/100
	Jaccard	0.932	0.931	0.948	0.942	0.952	0.057	0.068	1.813
	*N*/*C*	30/90	30/20	20/40	20/40	30/20	20/40	30/20	20/10
	Roger-Tanimoto	0.931	0.932	0.958	0.949	0.946	0.050	0.067	1.756
	*N*/*C*	40/60	10/50	30/10	30/10	10/50	30/10	10/50	30/70
	Sokal-Michener	0.932	0.946	0.959	0.949	0.960	0.050	0.053	1.793

ACC, accuracy; SEN, sensitivity; SPE, specificity; PPV, positive predictive value; NPV, negative predictive value; FDR, false discovery rate; FNR, false negative rate.

**Table 8:** Combinations *N*/*C* (*N* is the test size in percentage, and *C* is the parameter that controls the penalty for classification errors) that result in the best possible performance for our proposal on the subset of testing data.

**Dataset**	**Combination**	**Performance measure**							
	**Distance**	**ACC**	**SEN**	**SPE**	**PPV**	**NPV**	**FDR**	**FNR**	**Lift**
Cleveland HD	*N*/*C*	90/20	60/20	60/70	60/70	60/20	60/70	60/20	60/80
	Jaccard	0.927	0.963	0.957	0.948	0.966	0.051	0.036	1.856
	*N*/*C*	60/90	60/50	60/90	60/90	60/50	60/90	60/50	60/40
	Roger-Tanimoto	0.960	0.946	0.989	0.987	0.959	0.012	0.053	1.833
	*N*/*C*	60/60	60/90	60/20	60/20	60/90	60/20	60/90	90/80
	Sokal-Michener	0.927	0.953	0.980	0.971	0.953	0.028	0.046	1.416
Divorce	*N*/*C*	70/80	70/80	70/80	70/80	70/80	70/80	70/80	60/80
	Jaccard	1.000	1.000	1.000	1.000	1.000	0.000	0.000	2.125
	*N*/*C*	60/50	60/50	60/50	60/50	60/50	60/50	60/50	60/10
	Roger-Tanimoto	1.000	1.000	1.000	1.000	1.000	0.000	0.000	2.170
	*N*/*C*	70/80	70/80	70/80	70/80	70/80	70/80	70/80	60/50
	Sokal-Michener	1.000	1.000	1.000	1.000	1.000	0.000	0.000	2.081
Spambase	*N*/*C*	60/30	90/80	90/80	90/80	90/80	90/80	90/80	90/100
	Jaccard	0.965	1.000	1.000	1.000	1.000	0.000	0.000	1.749
	*N*/*C*	60/80	70/90	60/80	60/80	70/90	60/80	70/90	90/60
	Roger-Tanimoto	0.950	0.928	0.969	0.950	0.954	0.049	0.071	1.479
	*N*/*C*	90/60	70/80	90/50	60/40	60/80	60/40	70/80	60/100
	Sokal-Michener	0.926	0.934	0.934	0.950	0.957	0.049	0.065	1.561
Rice	*N*/*C*	60/20	80/80	90/50	60/40	60/20	60/40	80/80	70/90
	Jaccard	0.887	0.885	0.905	0.925	0.857	0.074	0.114	2.069
	*N*/*C*	60/30	80/50	60/20	60/20	60/30	60/20	80/50	80/10
	Roger-Tanimoto	0.888	0.885	0.907	0.926	0.853	0.073	0.114	2.067
	*N*/*C*	60/70	60/70	60/30	60/30	60/10	60/30	60/70	60/70
	Sokal-Michener	0.890	0.883	0.910	0.928	0.850	0.071	0.116	2.086
Banknote	*N*/*C*	60/80	70/50	60/80	70/80	70/50	70/80	70/50	60/70
	Jaccard	0.947	0.942	0.960	0.950	0.955	0.049	0.057	1.722
	*N*/*C*	60/90	60/90	60/80	70/80	60/90	70/80	60/90	80/50
	Roger-Tanimoto	0.946	0.945	0.929	0.944	0.955	0.055	0.054	1.721
	*N*/*C*	60/40	80/60	60/70	60/70	80/60	60/70	80/60	60/10
	Sokal-Michener	0.949	0.932	0.966	0.958	0.955	0.041	0.055	1.736

Conventions as in Table 7.

Table 9 shows the combinations of data set, distance, test size and value of *C* as well as the performance metrics when the maximum accuracy is achieved. For the Cleveland HD dataset the maximum precision is 96%, and occurs when *N* = 60, *C* = 90 and the Rogers-Tanimoto distance is used. For this combination of parameters, the sensitivity, specificity, PPV, NPV, FDR, FNR and lift are 93, 98.9, 98.7, 93.8, 1.2, 6.9, and 1.803, respectively.

**Table 9:** Combinations distance, *N* and *C* that result in the best accuracy.

**Dataset**	**Distance**	**N/C**	**Performance measure**							
			**ACC**	**SEN**	**SPE**	**PPV**	**NPV**	**FDR**	**FNR**	**Lift**
Cleveland HD	Jaccard	60/20	0.927	0.963	0.896	0.887	0.966	0.112	0.003	1.765
	Roger-Tanimoto	60/90	0.960	0.930	0.989	0.987	0.938	0.012	0.069	1.803
	Sokal-Michener	60/60	0.927	0.906	0.942	0.918	0.933	0.081	0.093	1.566
Divorce	Jaccard	70/80	1.000	1.000	1.000	1.000	1.000	0.000	0.000	1.983
	Roger-Tanimoto	60/50	1.000	1.000	1.000	1.000	1.000	0.000	0.000	2.125
	Sokal-Michener	60/20	1.000	1.000	1.000	1.000	1.000	0.000	0.000	1.888
Spambase	Jaccard	60/30	0.965	0.986	0.996	0.994	0.990	0.005	0.013	1.647
	Roger-Tanimoto	60/80	0.950	0.920	0.969	0.950	0.950	0.049	0.079	1.510
	Sokal-Michener	60/80	0.953	0.933	0.966	0.947	0.957	0.052	0.066	1.547
Rice	Jaccard	60/20	0.887	0.884	0.890	0.911	0.857	0.088	0.115	2.006
	Roger-Tanimoto	60/30	0.888	0.885	0.893	0.917	0.853	0.082	0.114	2.042
	Sokal-Michener	60/70	0.890	0.883	0.900	0.925	0.847	0.074	0.116	2.086
Banknote	Jaccard	60/80	0.947	0.930	0.960	0.944	0.950	0.055	0.069	1.614
	Roger-Tanimoto	60/50	0.946	0.931	0.957	0.942	0.949	0.057	0.068	1.624
	Sokal-Michener	60/40	0.949	0.932	0.962	0.952	0.946	0.047	0.067	1.695

Conventions as in Table 7.

For the Divorce dataset, the maximum precision is 100%, and occurs in different combinations of distance, test size and *C*; this result has been previously achieved by other authors (Juarez-Lopez et al., 2021). With the Spambase dataset, the maximum precision is 96.5% and occurs when *N* = 60, *C* = 30 and the Jaccard distance is used. Under these settings, the sensitivity, specificity, PPV, NPV, FDR, FNR and lift are 98.6, 99.6, 99.4, 99, 0.5, 1.3, and 1.647, respectively. When testing our approach with the Rice data set, the maximum accuracy achieved was 89%, and occurs when *N* = 60 and *C* = 70 and the Sokal-Michener distance is used. The sensitivity, specificity, PPV, NPV, FDR, FNR and lift performance measures at this set up were 88.3, 90, 92.5, 84.7, 7.4, 11.6, and 2.086, respectively. Finally, in the Banknote dataset, the maximum achieved accuracy was 94.9% when *N* = 60 and *C* = 40, and Sokal-Michener distance is used. In this case, sensitivity, specificity, PPV, NPV, FDR, FNR and lift are 93.2, 96.2, 95.2, 94.6, 4.7, 6.7, and 1.695, respectively. In general, these results indicate that the proposed method produces remarkable performance with accuracy, sensitivity, specificity, PPV and NPV above 84%, FDR and FNR values below 12% and lift values > 1.5 for the set of test data.

### 5.1 Comparison with other kernels

A comparative analysis assessing the performance of the proposed kernel against other kernels known in the literature was performed. Experiments were conducted setting *N* = {60%, 70%, 80%, 90%} and *C* = {10, 20, 30, 40, 50, 60, 70, 80, 90, 100} using different kernels. Table 10 shows the main results for the Cleveland HD dataset.

**Table 10:** Best accuracy obtained when different kernels for the Cleveland HD dataset.

**Kernel**	**Distance**	**N/C**	**Performance measures**							
			**ACC**	**SEN**	**PE**	**PPV**	**NPV**	**FDR**	**FNR**	**Lift**
Proposed	Jaccard	60/20	0.927	0.963	0.897	0.888	0.967	0.112	0.037	1.765
	Roger-Tanimoto	60/90	0.961	0.930	0.989	0.988	0.939	0.012	0.070	1.804
	Sokal-Michener	60/60	0.927	0.907	0.942	0.919	0.933	0.081	0.093	1.567
Linear	Jaccard	60/40	0.899	0.901	0.898	0.880	0.917	0.120	0.099	1.640
	Roger-Tanimoto	60/20	0.894	0.852	0.934	0.926	0.867	0.074	0.148	1.691
	Sokal-Michener	70/10	0.889	0.873	0.906	0.899	0.881	0.101	0.127	1.716
RBF	Jaccard	60/100	0.939	0.959	0.925	0.897	0.970	0.103	0.041	1.591
	Roger-Tanimoto	60/70	0.927	0.889	0.959	0.947	0.913	0.053	0.111	1.646
	Sokal-Michener	60/70	0.944	0.951	0.939	0.928	0.958	0.072	0.049	1.730
Sigmoid	Jaccard	90/50	0.873	0.841	0.901	0.883	0.865	0.117	0.159	1.600
	Roger-Tanimoto	70/30	0.846	0.816	0.873	0.851	0.842	0.149	0.184	1.553
	Sokal-Michener	90/10	0.858	0.836	0.877	0.850	0.865	0.150	0.164	1.539
Polynomial	Jaccard	60/40	0.927	0.944	0.910	0.914	0.942	0.086	0.056	1.902
	Roger-Tanimoto	60/100	0.939	0.951	0.929	0.917	0.958	0.083	0.049	1.727
	Sokal-Michener	60/90	0.922	0.905	0.937	0.927	0.918	0.073	0.095	1.710

RBF, radial basis function. The degree of the polynomial kernel function was *d* = 3. Conventions as in Table 7.

Overall, the accuracy, sensitivity, specificity, PPV and NPV values above 80%, FDR and FNR values below 18% and lift values > 1.5 for the dataset. However, the proposed method obtained the higest accuracy at 96.1% when the 60% test size is used, *C* = 90 and the Rogers-Tanimoto distance is utilized. For this combination of parameters, the sensitivity, specificity, PPV, NPV, FDR, FNR and lift are 93, 98.9, 98.8, 93.9, 1.2, 7.0 and 1.804, respectively.

**Figure 2:**
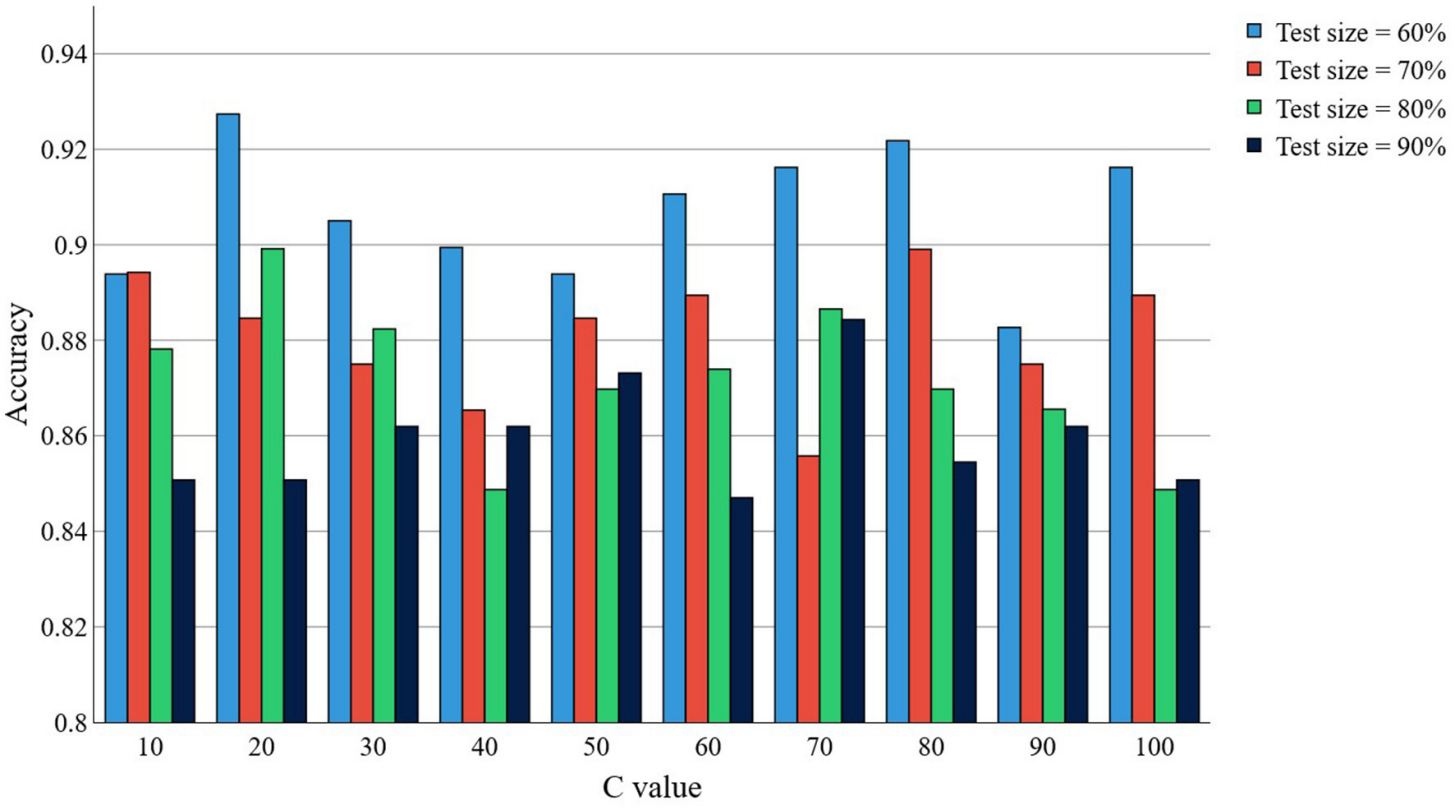
Accuracy for the Cleveland HD dataset, when using our proposed kernel, as a function of the *C* and “test size” parameters.

To determine the influence of the experiment parameters on the achieved accuracy for the Cleveland HD data set, an Analysis of Variance (ANOVA) was conducted. The effects of the parameter “kernel,” “distance,” “*C*, ” and “test size”, as well as their two-interactions were evaluated. Our results suggest that factors “kernel” (*P* < 2.2 × 10^−16^), “test size” (*P* < 2.2 × 10^−16^), the interaction between “kernel” and “test size” (*P* < 2.2 × 10^−16^), and the interaction between “kernel” and “*C*” (*P* = 0.00683) statistically significantly contribute to the achieved accuracy. This, overall, indicates that the selection of the kernel, the test size and the *C* parameter in SVM have a significant impact on the achieved accuracy. Interestingly, we found that the “*C*” parameter alone is not statistically significant (*P* = 0.232). Figure 2 depicts the accuracy results for the proposed kernel in the Cleveland HD dataset when the parameters *C* and *N* are changed. Note that optimal accuracy is primarily achieved when *N* decreases.

### 5.2 Comparison with similar studies

Table 11 shows the comparison of our proposal with other approaches in the literature using previously published data sets (Table 4). To the best of our knowledge, a detailed configuration of all experiments does not exist in the literature for all cases. Hence, here we use the accuracy for comparison purposes. Overall, our method performs reasonably well in all datasets compared to other approaches in the literature. In particular, our method outperforms other approaches using the Cleveland HD, Divorce and Spambase datasets, and achieves competitive performance with accuracy values above 89% for the Rice and Banknote datasets (Table 11). Interestingly, our method reaches this level of performance while using the proportion 40/60 for the training and testing data sets. This result could be or remarkable importance, especially when training SVM models in large datasets.

**Table 11:** Accuracy comparison of our proposal and other approaches in the literature using the previous datasets.

**Dataset**	**References**	**Method**	**Accuracy**
Cleveland HD	Verma et al., 2016	Ensemble of Majority vote with Naive Bayes, Bayes Net, Random Forest, and Multi perceptron	85.48%
	Verma et al., 2016	Hybrid model [multi-layer perceptron (MLP), multinomial logistic regression (MLR), fuzzy unordered rule induction algorithm (FURIA) and C4.5] with CFS (correlation-based feature subset) and PSO (particle swam optimization) feature selection.	90.28%
	Subhadra and Vikas, 2019	MLP neural network	93.39%
	Alotaibi, 2019	Decision tree	93.19%
	Khan et al., 2017	SVM	84.12%
	This proposal (40% training)		96.08%
Divorce	Sharma et al., 2021	Perceptron (60–40 split)	98.53%
	Simanjuntak et al., 2020	Backpropagation neural netwok (BPNN)	99.41%
	Juarez-Lopez et al., 2021	C4.5 (2/3 for training, 1/3 for testing)	95.60%
	Juarez-Lopez et al., 2021	KNN	100%
	This proposal (40% training)		100%
Spambase	Awad and Foqaha, 2016	Combination of radial basis function neural networks (RBFNN) and particles swarm optimization (PSO).	91.4%
	Ghosh and Senthilrajan, 2023	Random forest	99.93%
	Lee et al., 2010	Random forest	95.00%
	This proposal (40% training)		96.5%
Rice	Ilhan et al., 2021	Deep neural networks	93.04%
	Ilhan et al., 2021	Logistic regression	93.02%
	This proposal (40% training)		89.06%
Banknote	Yadav et al., 2021	Decision tree (80% training)	99%
	Yadav et al., 2021	Naive Bayes (70% training)	83.1%
	Yadav et al., 2021	Logistic regression (70% training)	99%
	Yadav et al., 2021	SVM (60% training)	98%
	This proposal (40% training)		94.9%

### 5.3 Multiclass classification

Table 12 shows the results of applying our approach to the Cleveland HD dataset without binarizing the class target variable, leaving a total of five classes (Janosi et al., 1989). However, the attributes are binarized following the approach previously mentioned in Section 3, since a binary matrix is required for the calculation of the dissimilarity matrix in our proposed method.

**Table 12:** **(A)** Confusion matrix for the Cleveland HD dataset when using the proposed method. **(B)** Performance metrics obtained at this configuration.

**(A) Confusion matrix**							
	**Real class**						
**Predicted**		**0**	**1**	**2**	**3**	**4**	
0		102	0	0	0	0	
1		0	27	1	2	0	
2		0	5	14	2	0	
3		0	1	4	13	0	
4		0	1	2	2	3	
**(B) Performance metrics**							
**ACC**	**SEN**	**SPE**	**PPV**	**NPV**	**FDR**	**FNR**	**LIFT**
1.000	1.000	1.000	1.000	1.000	0.000	0.000	2.325
0.942	0.900	0.953	0.794	0.979	0.206	0.100	0.980
0.928	0.670	0.955	0.667	0.956	0.333	0.333	0.755
0.944	0.720	0.962	0.684	0.969	0.316	0.278	0.765
0.974	0.380	1.000	1.000	0.972	0.000	0.625	1.017

(A) Here, *N* = 60% and *C* = 20 and the kernel is based on the Jaccard distance. (B) Conventions as in Table 7.

According to our results, the proposed method yields an overall accuracy of 89.39% in the testing dataset (Table 12A). This occurs when *N* = 60%, *C* = 20 and the Jaccard distance used as kernel. In this same combination of parameters, the average the sensitivity, specificity, PPV, NPV, FDR, FNR and lift for all classes are 0.734, 0.974, 0.829, 0.975, 0.171, 0.267, and 1.169, respectively. These multiclass classification results are satisfactory since there is a high probability of correctly identifying positive and negative cases. Interestingly, our proposed method showed superior performance for the binary classification (96.08% accuracy, Table 9) than for multiclass classification (89.39%), which is comparable with other results in the literature.

## 6 Conclusions and future work

Here we present and illustrate an innovative distance-based kernel for binary classification using SVMs as well as an iterative procedure to identify the best training/testing data sets combination maximizing the accuracy. We also showed that our approach is easily extended to multiclass classification situations. The effectiveness of the classification approach is evaluated through variations in parameters, and the use of four data sets with different number of instances and features. When evaluating the effectiveness of our approach, we conducted comparative analyses against prevalent methodologies documented in the literature. Remarkably, even without selectively choosing specific attributes, our method exhibited comparable, and, in certain instances, superior performance than established approaches previously published. These findings validate the credibility and effectiveness of our proposed method. Moreover, the statistical significance of the kernel as a determining factor in result quality became evident after comparing several kernels. By tackling both binary and multiclass classification tasks, our model exhibited an outstanding ability to handle both modalities successfully, thus highlighting its versatility and proficiency in a variety of classification scenarios.

The main contributions of our work are: (*i*) we describe in detail an algorithm to determine the best subset of the original dataset to represent the data and fit the model; (*ii*) propose a novel distance-based kernel method, implement it in a SVM, and apply it to well-known publicly available datasets (Janosi et al., 1989; Mark Hopkins, 1999; Lohweg, 2012; Koklu and Cinar, 2019b; Mustafa Yntem, 2019) achieving remarkable performance; and (*iii*) conduct computational experiments with such data sets and show that our proposal overcomes, in terms of performance, other kernel methods and classification models available in the literature.

As part of future research perspectives, three key areas are proposed. First, it is essential to determine the performance of the current model through a careful feature selection process. This will allow the identification of the most relevant variables and will contribute to a greater efficiency and precision in classification problems. Secondly, the implementation of a specific improvement process for the existing multiclass classification system should be assessed. This involves algorithm optimization, hyperparameter tuning, and continuous evaluation to ensure optimal performance in multiclass scenarios. Finally, we strongly suggest expanding our proposal toward an unsupervised classification system. This would open the door to automatically identify emerging patterns and structures in the data, which could have applications in the exploration of complex data sets, and subsequently detect potential anomalies. These research directions represent crucial steps to advance the efficiency and versatility of classification models in the current context of data analysis.

## Data availability statement

Publicly available datasets used in this study can be retrieved from the following URLs: https://archive.ics.uci.edu/dataset/45/heart+disease. Divorce dataset: https://archive.ics.uci.edu/dataset/539/divorce+predictors+data+set, Spambase dataset: https://archive.ics.uci.edu/dataset/94/spambase, Banknote dataset: https://archive.ics.uci.edu/dataset/267/banknote+authentication, Rice dataset: https://archive.ics.uci.edu/dataset/545/rice+cammeo+and+osmancik.

## Author contributions

NA-T: Conceptualization, Formal analysis, Software, Writing – original draft. MG: Formal analysis, Supervision, Writing – original draft. JV: Conceptualization, Formal analysis, Writing – review & editing. EZ: Conceptualization, Writing – review & editing.
